# Point for Enrichment, Point for Welfare—Testing Use of a Laser Pointer with *Arapaima gigas*

**DOI:** 10.3390/ani13081370

**Published:** 2023-04-17

**Authors:** Eszter Matrai, Hau Yin Alfred Chan, Fung Ming Leung, Shaw Ting Kwok, Xiao Lin, Paolo Martelli

**Affiliations:** 1 Research Department, Ocean Park, Hong Kong 180 Wong Chuk Hang Road, Aberdeen, Hong Kong; 2 Department of Ethology, Eötvös Loránd University, Pázmány Péter Sétány 1/c, H-1117 Budapest, Hungary; 3 Department of Psychiatry, Icahn School of Medicine at Mount Sinai, New York, NY 10029-5674, USA; alanlamsiu@gmail.com

**Keywords:** fish enrichment, pirarucu, arapaima behavior, environmental enrichment, activity monitoring

## Abstract

**Simple Summary:**

Environmental enrichments are essential tools for providing appropriate welfare conditions for animals under human care by stimulating mental and physical activity. Enrichment programmes in zoos and aquariums have developed rapidly over the past two decades, especially for charismatic mammal and bird species. However, when it comes to lesser-known species such as the arapaima (*Arapaima gigas*), there is still room for improvement. The arapaima is one of the largest freshwater fish species. It is fished and farmed for its meat but also kept on display in Asian aquariums. Despite its agricultural and ecological importance, there is limited information on the behaviour of the species. Its conservation status on the IUCN Red List continues to be “Data Deficient”. In this study, we investigate the use of a green laser pointer as a potential visual enrichment for arapaimas. Without displaying any aggression or territorial behaviour, the fish were more active and utilized more of their habitat in the presence of the novel stimulus. We believe that our findings support the use of the laser pointer as environmental enrichment for arapaimas and provide a basis for further behavioural and welfare research with the species.

**Abstract:**

The arapaima (*Arapaima gigas*) is one of the largest freshwater fish species, known to exceed 3 m in total length. It is listed as Data Deficient by the IUCN. *A. gigas* is native to the Amazon River basin where they are an important food source. Arapaimas are also farmed for meat and for live specimens in various South American and Asian countries. Despite decades of keeping the species in public aquariums, little is known of its behaviour and cognitive abilities. This pilot study provides baseline data on using a green laser pointer as environmental enrichment for this species under human care. The data collection included 18 observations before the use of the laser pointer (baseline) and 18 observations during the use of the laser pointer (test). Ten behaviours were monitored, investigating physical contact, activity pattern and habitat use by the fish. During the test, the fish significantly increased their presence in the tank, their level of activity and their use of the habitat. This pilot study provides valuable baseline data for further investigations demonstrating the value of a laser pointer as environmental enrichment for *A. gigas* under human care.

## 1. Introduction

The arapaima (*Arapaima gigas*; Schinz, 1822) is endemic to the Amazon and Essequibo basins of South America [1]. Arapaimas are commonly displayed in public aquariums due to their spectacular size. Adult specimens can grow up to 4.5 m in length and 200 kg in weight [2]. Their numbers in the wild are unknown and they are threatened by overfishing, habitat degradation, by-catch of juveniles, and recreational angling [3,4,5]. The species is listed in Appendix II of the Convention on International Trade of Endangered Species of Wild Fauna and Flora (CITES), yet continues to be “Data Deficient” in the IUCN Red List [6]. Therefore, specimens kept in aquarium collections may play an important role in the conservation of the species. A better understanding of the behaviour and the welfare requirements of the species is essential not only for adequate zoological management of the species but also for the future of successful breeding programmes.

Assessing fish welfare has become more critical in the last decades. The welfare aspects of fishes and other aquatic animals are poorly developed compared to terrestrial taxa due to the broad diversity of aquatic species and difficulties in assessing behavioural patterns [7]. As ethology has evolved from descriptive studies into a meticulous science incorporating sophisticated experimental manipulations and data analyses, well-developed ethograms provide the basis for valid and efficient experimentation [8]. The establishment of basic ethograms is essential for behavioural analysis. While there is a lack of behavioural studies for arapaimas, there is increasing literature on both freshwater and seawater fishes. For example, Bolhan and her colleagues documented the behavioural repertoire of Arctic charr (*Salvelinus alpinus*) under human care by establishing a species-specific ethogram with behaviours grouped into four categories: locomotion, stationary positions, social interaction and displays, and feeding [9]. In another study, Branconi and her colleagues focused on the differences in live and video coding of Humbug damselfish (*Dascyllus aruanus*) behaviour. Their detailed ethogram included 36 different behaviours that were classified into five categories such as aggressive, social, maintenance, reproductive, and submissive [10]. Ethograms have also been adapted to investigate social plasticity [11], genotype-environment interaction [12], play behaviour [13], or even behavioural responses to interactive robots [14,15].

Environmental enrichment plays an important role in the welfare of animals under human care (e.g., [16,17,18]). It provides both mental and physical stimulation, reduces boredom and stereotypy, and ultimately contributes to the health of the animals [18,19,20]. Environmental enrichments for fish represent a developing field and currently, only a handful of studies have been published. Lee and Berejikian investigated steelhead (*Oncorhynchus mykiss*) adaptive behaviours by providing them with the choice of two tanks, one of which was barren and the other was enriched with rocks and plastic plants. They documented changes in average behaviour and behavioural variation in response to the differences in the environment [17]. In other studies, structural enrichment was also documented to positively influence weight gain in juvenile rainbow trout (*Oncorhynchus mykiss*) [21], and improved behavioural flexibility and learning ability were improved in juvenile Atlantic salmon (*Salmo salar*) [22]. Another study found that the affiliative behaviour in zebrafish (*Danio rerio*) increased when novel space was present for them to explore [20]. A meta-analysis of 1171 reviewed studies on the impact of physical enrichment on aquatic animals under human care showed a significant positive welfare effect while under human care and post-release [23]. In another study, the introduction of black rockfish (*Sebastes schlegelii*) increased growth in greenling (*Hexagrammos otakii*). Thus, adding the second species is considered an effective social enrichment [24]. The same team also presented black rockfish (*Sebastes schlegelii*) with enriched vs. barren environments. The fish from the barren environment were observed to exhibit increased aggressive behaviour [25]. Structural environmental enrichment and tryptophan effectively reduced stereotypical and aggressive behaviour in Nile tilapia [26].

Light provides essential environmental cues for fish [27] and plays a crucial role in their behavioural ecology (e.g., [28,29]). Artificial light was regarded as a behavioural guidance tool for fish, mainly used to increase catch yield in fisheries. Fires on the beach and LED lights on the fishing gear attracted fish at night [30,31,32,33]. Artificial light was also used to deter the movement of fish, and lower fish mortality during the construction of hydraulic dams by guiding them away from the dangerous place and towards the replacement habitat [34]. Moreover, artificial light has been commonly used for behavioural ecology research. Behavioural response to light exhibited by different fish species showed a correlation with the species’ diel activity patterns: the diurnal fishes preferred illuminated areas, whereas the nocturnal/crepuscular fishes preferred darkened areas [28]. In another study, artificial light was used to enhance the passage of juvenile salmonids at Bonneville Dam [35].

Consequently, the use of light can play a vital role in designing environmental enrichments for fish [36]. Laser pointers could serve as a tool for light-based environmental enrichment. During a benthic trawl in Lyme Bay, fish were found to be attracted to a laser pointer and exhibited agonistic behaviour [37]. However, in a recent study, 66 fish species were exposed to a laser pointer. It was found that the majority of the fish showed at least a moderate level of interest in the stimuli [13].

In light of the above, we investigated the use of a green laser pointer as a potential visual enrichment for our three arapaimas at Ocean Park Hong Kong. We hypothesised that the successful use of the laser pointer would attract the fish, engage them in increased locomotive activity and motivate them to utilise more areas of the habitat.

## 2. Materials and Methods

### 2.1. Subject and Housing

**Figure 1:**
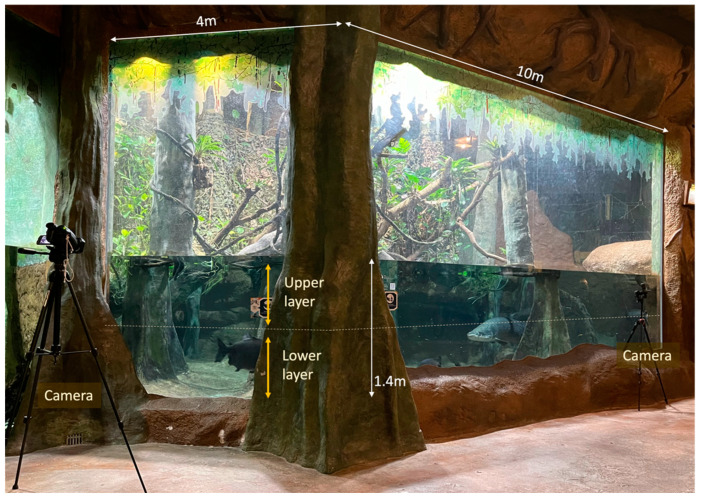
The research setup at the habitat (exhibition tank only) of the three *Arapaima gigas*, the dashed line indicates the border between the upper and lower layer of the water body.

The study subjects were three arapaimas (*Arapaima gigas*) living in the Expedition Trail of the Rainforest exhibition of Ocean Park Hong Kong. The demographic information of the three fish is summarised in Table 1. The arapaimas were housed in two interconnecting freshwater tanks along with other species, including a redtail catfish (*Phractocephalus hemioliopterus*), two alligator gars (*Atractosteus spatula*), and four pacus (*Colossoma brachypomum*). The habitat included one exhibition tank (W 4 m × L 10 m × D 1.4 m, Figure 1) and one back-of-house tank (W 2.4 m × L 5.2 m × D 1.4 m). The data collection focused on the exhibit tank exclusively; the *A. gigas* were free to move between two tanks for the entire study period. They were fed once a week with 0.75 kg of mackerel (*Scombridae japonicus*) and bonito (*Euthynnus affinis* and *Katsuwonus pelamis*). They received no specific training and were not included in scientific research before this study.

### 2.2. Data Collection

The experiment was conducted twice weekly between November 2019 and October 2020, excluding two lockdown periods (February–March and July–August 2020) due to COVID-19. Each research session lasted 15 min. The timing of the sessions was selected in a pseudo-random manner, between 09.00 and 18.00, with observations conducted twice every hour for each condition (two observations at 9.00–9.15, two observations at 10.00–10.15, etc.). Thus, the experiment included 18 baseline (control) and 18 test sessions. As the fish had no known daily activity peaks, we developed the above methodology to cover the daily behaviour of the fish during the working days.

The data collection was conducted using BORIS software [39]. The sessions were coded using all occurrence sampling [40] and focal following. The ethogram included ten behaviours, investigating activity, habitat use, and physical contact (Table 2 and Table S1). All ten behaviours were coded for frequency and seven for frequency and duration (state events). Before the experiment, each analyser was required to complete the coding and individual identification training successfully. New analysers were paired with experienced ones during the training. New analysers received qualification if they achieved high inter-rater reliability (Cohen’s Kappa ≥ 0.60) on three consecutive practice sessions.

Two GoPro Hero6 Black action cameras and a Sony FDR-AX100 4K Handycam were used for high-quality video data collection (Figure 1). The footage was reviewed during post-session verification.

### 2.3. Procedure

The testing sessions were conducted using a Logitech professional presenter R800 (laser pointer). The laser pointer was used in the upper and lower layers of the water body equally (Figure 1). Targets included tree trunks, the stone background wall, the floor, and the sitting area in front of the windows. The laser pointer was used at each location for 4–5 s before moving on to the next one. To prevent any potential harmful effect on the fish, the pointer was turned off between locations and was not used closer than 30 cm to the animals’ eyes (including all fish in the tank, not only the three *A. gigas*).

### 2.4. Data Analysis

The three fish, two males (Aron and Casmir) and one female (Beth) were free to move between the two tanks for the entire observation period, hence their presence during the sessions was considered voluntary participation. During the observations, there was always at least one of the three arapaimas in the exhibition tank. The complete absence of an individual was still included in the analysis with zero values assigned. The frequency and duration of the behavioural units (Table 2) were compared between baseline and test conditions. If the laser pointer was proven to be an appropriate environmental enrichment, we expected to see an increase in travelling vs. station holding and increased habitat use.

Because the fish were not always present in the exhibition tank (out of view), the data size was unequal for the three individuals, relative durations and relative frequencies were also calculated for the analysis. The relative duration values were calculated by dividing the times a fish spent on one behaviour during one session by the total time he/she was visible during that session (i.e., the time spent swimming by Aaron during the first baseline session divided by the total time he was visible in the exhibit pool during that session). Relative frequencies were calculated by dividing the number of times a fish performed one behaviour in one session by the total time he/she was visible during that session (i.e., the number of times Aaron was recorded to engage in swimming during the first baseline session divided by the total time he was visible in the exhibit pool during that session). The calculation of the relative frequencies relies on the assumption that there is a linear relationship between time and frequency, providing a certain degree of limitation to our analysis. However, it allowed a better evaluation of the changes in frequency in response to the novel stimulus rather than reflecting the time the fish spent in the exhibition tank. Both observed and calculated relative values were compared for baseline vs. test conditions.

The change of location between the upper and lower layer of the waterbody was also calculated and compared for baseline vs. test conditions. The R (R version 4.2.2) and lme4 [41] were used to perform a linear mixed effects analysis of the relationship between the presence of laser pointer and different measures of behaviours. The presence of the laser pointer was set as a fixed effect and the individual fish was set as an intercept random effect. Visual inspection of the Q-Q plot revealed no obvious deviations from normality. Hypothesis testing was performed by likelihood ratio tests of the full model with the laser pointer against the model without the laser pointer.

## 3. Results

**Figure 2:**
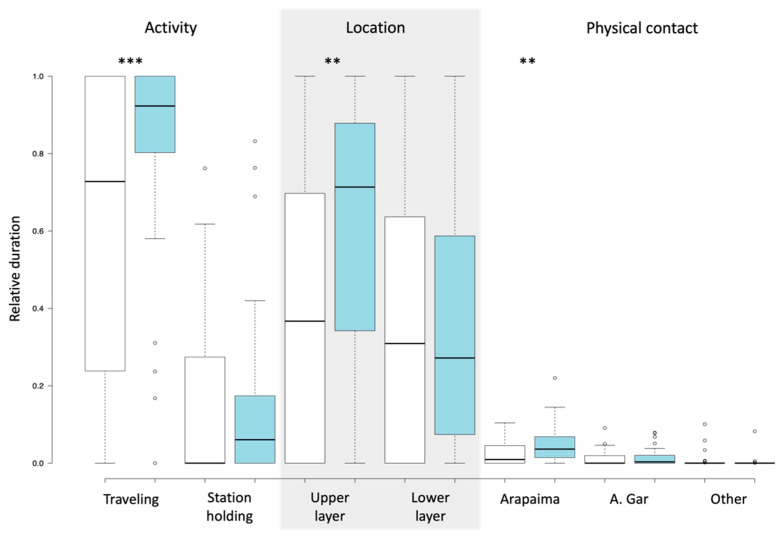
The relative duration (observed duration/fish presence in the exhibit tank) of the seven monitored state behaviours (behaviour where duration was documented); during the test (blue) and baseline (white) conditions. Boxplots represent the median and interquartile range (IQR) with whiskers indicating the top and bottom 5% of occurrences and outliers are represented by the circles. Significant change between baseline and test conditions is marked with asterisks (** *p* > 0.01, *** *p* > 0.001).

During test sessions, two of the three fish were more frequently visible. Aaron appeared in 17/18 (baseline) vs. 13/18 (test) sessions, Beth appeared in 17/18 (baseline) vs. 10/18 (test) sessions, while Casmir was always present. The three fish also spent more time in the exhibit tank during the test (average duration ± SD: 50% ± 42% vs. 63% ± 35%). The comparison of the observed and the relative duration values showed similar results. The three fish showed an increased level of activity: during the test the fish spent more time travelling (average relative duration ± SD: 62% ± 39% vs. 83% ± 25%), using the upper layer of the waterbody (40% ± 36% vs. 60% ± 34%), and engaging in physical contact with conspecifics (3% ± 3% vs. 5% ± 5%; all *p* < 0.05; Figure 2; Table 3 and Table 4).

**Figure 3:**
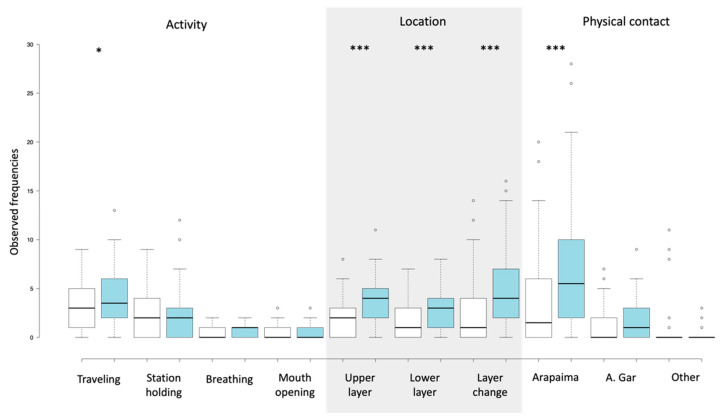
The observed frequency of the ten monitored behaviours during the test (blue) and baseline (white) conditions. Boxplots represent the median and interquartile range (IQR) with whiskers indicating the top and bottom 5% of occurrences and outliers are represented by the circles. Significant change between baseline and test conditions is marked with asterisks (* *p* > 0.05, *** *p* > 0.001).

**Figure 4:**
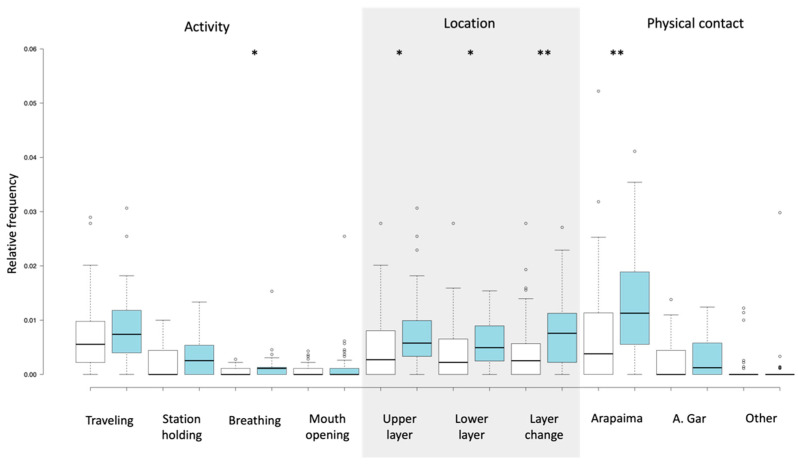
The relative frequency (observed frequency/fish presence in the exhibit tank) of the ten monitored behaviours during test (blue) and baseline (white) conditions. Boxplots represent the median and interquartile range (IQR) with whiskers indicating the top and bottom 5% of occurrences and outliers are represented by the circles. Significant change between baseline and test conditions is marked with asterisks (* *p* > 0.05, ** *p* > 0.01).

The observed and the calculated relative behavioural frequencies showed similar results to the behavioural duration analysis. The observed frequency of travelling, but not the relative frequency of travelling, increased significantly during test sessions (Table 5 and Table 6; Figure 3 and Figure 4). With increased activity, the use of both upper and lower layers and the change between the layers increased significantly, which was consistent for both observed and relative frequencies (all *p* < 0.05; Table 4 and Table 5, Figure 3 and Figure 4). Furthermore, with increased travelling, the relative frequency of breathing and both the observed and the relative frequencies of physical contact with conspecific also increased (all *p* < 0.05; Table 5 and Table 6; Figure 3 and Figure 4).

**Figure 5:**
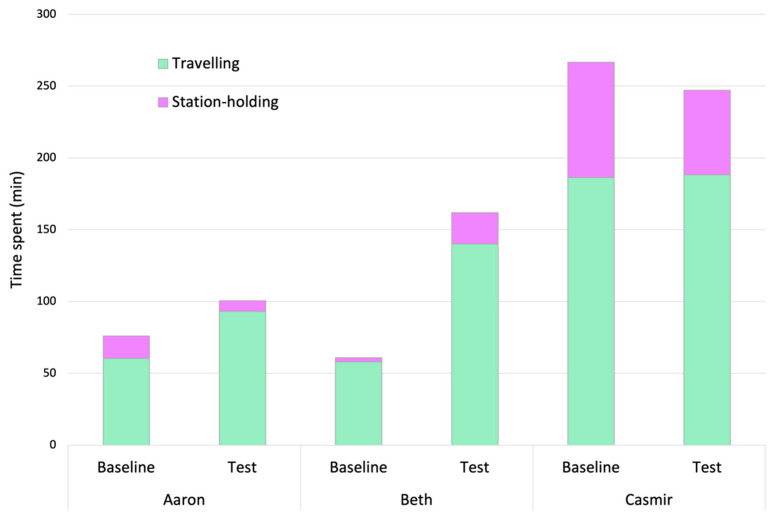
The observed duration of travelling (green) and station holding (purple) exhibited by the three *Arapaima gigas*, during baseline and test conditions; the cumulative duration of the two behaviours also indicates the presence of the three fish in the exhibit tank.

The documented changes were the most apparent in Beth’s behaviour (Figure 5). She spent more time travelling and in the upper layer during test sessions.

## 4. Discussion

This pilot study examined whether a laser pointer could be applied as an environmental enrichment for arapaimas under human care. The results demonstrated that during the use of the laser pointer, the fish increased their presence, activity, and habitat use, supporting the use of the novel enrichment.

With the laser pointer, the arapaima used the exhibit tank more frequently than the back-of-house tank. During the baseline phase, two of the three arapaimas (Aaron and Beth) often stayed inside the back-of-house tank. At least one was partially absent in all 18 baseline sessions and entirely absent in 11. The increased presence of the arapaimas in the exhibit tank was also apparent in the increased use of the upper layer of the water body during the test, while the time spent in the lower layer showed no difference between baseline and testing conditions. Thus, the increased time spent in the upper layer reflects the overall increased presence in the exhibit tank. Their tendency to stay off-exhibit (thus out of sight) could reflect on potential territorial behaviour [42,43,44]. It is also possible that the fish simply stayed at the back-of-house pool due to individual preference, or both. Nonetheless, when the laser pointer was in use, Aaron and Beth entered the exhibit tank more frequently and for a longer duration. There was no change in Casmir’s presence as it remained nearly equal between the baseline and the test session. It is important to mention that during the simultaneous presence of the three arapaimas in the exhibit tank, no inter- or intra-species aggression was documented. The laser pointer provided an attractive stimulus for the fish without creating social conflict. Similar to other studies, the introduction of novel enrichments increased and enhanced the animals’ use of their habitat [45,46,47]. Thus, we conclude that the positive change in the time spent present in the exhibit tank reflects the arapaimas’ interest towards the enrichment item and is considered a positive outcome for the fish and the visitors.

The laser pointer enrichments also enhanced activities, as indicated by increased travelling, layer changes, and physical contact during test sessions. Adequate environmental enrichments have been documented to increase exploratory behaviour in steelhead (*Oncorhynchus mykiss*, [17]), prompted attentiveness/tracking, chasing, and catching in fish [13], and increased activity is generally accepted as a positive welfare indicator [48]. Thus, the documented increased travelling further supports the positive effect of the laser pointer.

As the laser pointer was used equally between the upper and the lower layers of the waterbody, the fish were recorded not only travelling more but also changing between the layers more often than during baseline. Hence, the use of the laser pointer resulted in greater habitat use. Successful utilisation of novel environmental enrichments has been previously recorded to increase positive utilisation of the habitat in other species [7,20]. Therefore, the documented increase in layer change provides further support for the use of the laser pointer as environmental enrichment for arapaimas.

The observed difference in activity level between baseline and test conditions was the most prominent in Beth’s behaviour while it was least prominent in Casmir’s behaviour. We cannot exclude the possibility that individual differences may be responsible for the difference between the three arapaimas’ behaviour. Individuals may respond differently toward the same stimulus or environment even though they are the same species, the behavioural components of their response can be regarded as part of the animal’s personality [49]. However, as mentioned before, the differences could also reflect on the territorial behaviour [42,43,44] of the three fish. Thus, we modelled the variation of the same fish in repeated measurements as a random effect. As the laser pointer was used in the exhibition tank which was the preferred habitat of Casmir, the effect of the laser pointer was more prevalent in Aaron’s and Beth’s behaviour.

The three arapaimas spent more time at the exhibit tank and moved around in their habitat during test sessions which resulted in a higher chance of physical contact between the three fish and the other inhabitants of the tank. Only one laser pointer was used, and consequently, it is possible that the simultaneous interest of multiple fish might have led to the increase in physical contact. It was difficult to characterise finer movement patterns, such as target following, synchronous swimming, or leader-follow swimming due to the relatively slow movements of the fish and the design of the habitat. However, we cannot exclude the possibility of stimulus enhancement, attraction to the stimulus could be generated by the vicinity or by the interest of a conspecific [50]. Future studies in larger habitats, where fish have more room for movement and manoeuvrings could provide more in-depth data on the arapaima’s response.

While there has been an increase in the number of studies on environmental enrichment use with charismatic and flagship species under human care (e.g., [46,47]), there is certainly a need for investigations with lesser-known species such as the *A. gigas*. To our knowledge, this study is the first to explore the use of any environmental enrichment with arapaimas. The current literature on this species is limited to publications regarding its biology and farming. In line with the recent findings of other fish species’ response to the use of a laser pointer [13], our arapaimas’ response could be categorised as moderate or even high.

In conclusion, while our study only included three individuals in one habitat, we believe our data provide a valuable baseline for further investigations. We hope that our initial success will inspire other care facilities to replicate our study and build on it. Our results show positive changes in the behaviour of the three fish supporting the value of a laser pointer as environmental enrichment for arapaimas.

## Figures and Tables

**Table 1:** Demographic information of the three *Arapaima gigas* at Ocean Park Hong Kong.

Name	Sex ^1^	Date of Adoption ^2^	Origin
Casmir	Male	2011	Indonesia
Beth	Female	2011	Indonesia
Aaron	Male	2011	Indonesia

^1^ The sex of the fish was determined based on morphological characteristics after [38]. ^2^ The three arapaimas were estimated to be two years old when they arrived at Ocean Park.

**Table 2:** Operational definition of the observed behavioural factors, behaviours were defined as State events (with duration) or Point events (no duration).

Category	Behaviour	Type	Description
Activity	Travelling ^a^	State	Body parallel and moving relative to the substratum (fins moving), mutually exclusive with station holding
Activity	Station-holding ^a^	State	Stop movements for 10 s or longer, usually tail curls up, mutually exclusive with travelling
Activity	Breathing ^b^	Point	Surfaces and gulps of air
Activity	Mouth opening	Point	Mouth opening and closing underwater
Location	Upper layer	State	Majority of the fish’s body was in the upper half of the water body, mutually exclusive with lower layer
Location	Lower layer	State	Majority of the fish’s body was in the bottom half of the water body, mutually exclusive with upper layer
Location	Layer change ^b^	Point	Each even when the fish crossed from one layer to the other
Physical contact	Arapaima physical contact	State	Physical touch with another arapaima with any part of the body
Physical contact	Alligator gar physical contact	State	Physical touch with an alligator gar with any part of the body
Physical contact	Other physical contact	State	Physical contact with pacu or redtail catfish with any part of the body

^a^ Behavioural definition was based on the earlier work of Bogan et al., 2016 [22]. ^b^ Behavioural definition was based on the earlier work of Lennox et al., 2018 [17].

**Table 3:** Results of observed duration likelihood ratio test (df = 1) comparing the two linear mixed models, one with and the other without the fixed effect (effect of the use of the green laser pointer).

Behaviour	χ^2^(1)	*p*-Value
Travelling	7.878	0.005 *
Station-holding	0.213	0.645
Upper layer	10.924	<0.001 *
Lower layer	0.753	0.386
Arapaima physical contact	8.833	0.003 *
Alligator gar physical contact	0.303	0.582
Other physical contact	2.356	0.125

* *p*-values < 0.05 are considered significant.

**Table 4:** Results of relative duration (time spent with each behaviour divided by the time the individual spent in the exhibition tank) likelihood ratio test (df = 1) comparing the two linear mixed models, one with and the other without the fixed effect (effect of the use of the green laser pointer).

Behaviour	χ^2^(1)	*p*-Value
Travelling	10.964	<0.001 *
Station-holding	0.094	0.759
Upper layer	9.430	0.002 *
Lower layer	0.002	0.969
Arapaima physical contact	7.382	0.007 *
Alligator gar physical contact	0.458	0.499
Other physical contact	0.640	0.640

* *p*-values < 0.05 are considered significant.

**Table 5:** Results of observed frequency likelihood ratio test comparing the two linear mixed models, one with and the other without the fixed effect (effect of the use of the green laser pointer).

Behaviour	χ^2^(1)	*p*-Value
Travelling	5.671	0.017 *
Station-holding	0.956	0.328
Breathing	2.421	0.120
Mouth opening	0.016	0.899
Upper layer	11.349	<0.001 *
Lower layer	11.642	<0.001 *
Layer change	10.894	<0.001 *
Arapaima physical contact	12.530	<0.001 *
Alligator gar physical contact	1.402	0.236
Other physical contact	2.126	0.145

* *p*-values < 0.05 are considered significant.

**Table 6:** Results of relative frequency (observed frequency/observation duration) likelihood ratio test comparing the two linear mixed models, one with and the other without the fixed effect (effect of the use of the green laser pointer).

Behaviour	χ^2^	*p*-Value
Travelling	2.958	0.086
Station-holding	2.792	0.095
Breathing	5.032	0.025 *
Mouth opening	1.292	0.256
Upper layer	4.350	0.038 *
Lower layer	4.536	0.033 *
Layer change	8.149	0.004 *
Arapaima physical contact	8.190	0.004 *
Alligator gar physical contact	1.313	0.252
Other physical contact	0.006	0.939

* *p*-values < 0.05 are considered significant.

## Data Availability

Data is contained within the article or Supplementary Material. The data presented in this study are available in Supplementary Table S1.

## Supplementary Materials

The following supporting information can be downloaded at: https://www.mdpi.com/article/10.3390/ani13081370/s1, Table S1: Baseline and test data.
